# Step-wise approach to prevention of chronic diseases in the Danish primary care sector with the use of a personal digital health profile and targeted follow-up – an assessment of attendance

**DOI:** 10.1186/s12889-019-7419-4

**Published:** 2019-08-13

**Authors:** Lars Bruun Larsen, Jens Sondergaard, Janus Laust Thomsen, Anders Halling, Anders Larrabee Sønderlund, Jeanette Reffstrup Christensen, Trine Thilsing

**Affiliations:** 1 Department of Public Health, Research Unit of General Practice, University of Southern Denmark, J.B. Winsløws Vej 9A, 5000 Odense, Denmark; 2 Department of Clinical Medicine, Research Unit for General Practice, Aalborg University, Aalborg, Denmark; 3 Department of Clinical Sciences in Malmö, Centre for Primary Health Care Research, Lund University, Lund, Sweden

**Keywords:** Health promotion, Indicated prevention, Integrated prevention

## Abstract

**Background:**

Current evidence on chronic disease prevention suggests that interventions targeted at high-risk individuals represents the best way forward. We implemented a step-wise approach in the Danish primary care sector, designed for the systematic and targeted prevention of chronic disease. The intervention centered on a personal digital health profile for all participants, followed by targeted preventive programs for high-risk patients. The present paper examines individual characteristics and health-care usage of patients who took up the targeted preventive programs in response to their personal digital health profile.

**Methods:**

A sample of patients born between 1957 and 1986 was randomly selected from the patient-list system of participating general practitioners in two Danish municipalities. The selected patients received a digital invitation to participate. Consenting patients received a second digital invitation for a personal digital health profile based on questionnaire and electronic patient record data. The personal digital health profile contained individualized information on risk profile and personalized recommendations on further actions. If at-risk or presenting with health-risk behaviour a patient would be advised to contact either their general practitioner or municipal health centre for targeted preventive programs. Attendance at the targeted preventive programs was examined using Poisson regression and chi-squared automatic interaction detection methods.

**Results:**

A total of 9400 patients were invited. Of those who participated (30%), 22% were advised to get a health check at their general practitioner. Of these, 19% did so. Another 23% were advised to schedule an appointment for behaviour-change counselling at their municipal health centre. A total of 21% took the advice. Patients who had fair or poor self-rated health, a body mass index above 30, low self-efficacy, were female, non-smokers, or lead a sedentary lifestyle, were most likely to attend the targeted preventive programs.

**Conclusions:**

A personal digital health profile shows some promise in a step-wise approach to prevention in the Danish primary care sector and seems to motivate people with low self-efficacy to attend targeted preventive programs.

**Trial registration:**

Registered at Clinical Trial Gov (Unique Protocol ID: TOFpilot2016). Prospectively registered on the 29th of April 2016.

## Background

General health checks to prevent type-2 diabetes mellitus (T2DM) and cardiovascular disease (CVD) have shown no population-level effects above and beyond those attained by opportunistic case finding [1, 2]. However, it is well-known that as the absolute risk of CVD increases, so too does the protective effect of both health-behaviour change and preventive treatment [3–5]. This suggests that taking a more targeted approach to the prevention of chronic diseases, with a focus on high-risk individuals, may be warranted [6]. Advanced digital technology and infrastructure have only recently made such targeted approaches possible without putting too much strain on available human and economic resources. Most importantly, these advances may facilitate individual risk assessment using information already available in health-care databases or non-clinical health information. Examples of this added utility include the English National Health Services (NHS) in their health check program [7], as well as the Dutch cardio-metabolic disease (CMD) program [8].

Based on a technical feasibility study of a non-laboratory-based risk assessment from 2012 we designed the TOF (Danish acronym for Early Detection and Prevention) intervention [9]. TOF is a step-wise approach to systematic and targeted prevention of T2DM, CVD and chronic obstructive pulmonary disease (COPD), designed for the Danish primary health-care system. The intervention has two elements: a general and a targeted intervention. The general intervention involves the creation of personal digital health profiles for each individual in the entire study population. The targeted intervention involves a health check at the general practitioner (GP) or behaviour-change counselling at a municipal health centre (MHC). The targeted intervention applies only to those patients who were deemed likely to benefit from such intervention due to either their high, overall risk of the aforementioned diseases, or because they regularly engaged in health-risk behaviours. The TOF intervention was tested for acceptability, feasibility, and short-term effects in a large-scale pilot study during the period from April 2016 to December 2016 [10].

To our knowledge, only two studies have evaluated the uptake of similar, step-wise approaches (web-based risk assessment, risk-based referral) to preventive programs aimed at cardio-metabolic disease (CMD) or CVD [8, 11]. However, neither of the two studies reported on patient characteristics or health-care usage in the context of program attendance. In this study we therefore set out to examine attendance in targeted preventive programs in the Danish primary care sector in response to a personal digital health profile.

## Methods

### Design

A cross-sectional analysis of attendance at the targeted intervention of the TOF pilot study.

### Setting

The TOF pilot study took place in the municipalities of Varde and Haderslev from May 2016 to December 2016. The municipalities of Varde and Haderslev are two average-sized rural municipalities (approx. 50.000 inhabitants) in the Region of Southern Denmark. A total of 68 GPs resided in the two municipalities. Primary disease prevention such as smoking cessation, dietary advice, physical activity, and treatment of alcohol addiction falls under the responsibility of MHCs and is performed by qualified health professionals including nurses, dieticians, occupational therapists, and physiotherapists. Secondary disease prevention, such as preventive medical treatment and patient education in chronic disease management, is shared between the municipalities and the regional health authorities and is generally undertaken by GPs. Danish GPs are organised in publicly funded and privately-owned clinics with an average of two GPs per clinic. Each clinic employs a patient-list system with an average of 1600 patients per GP [12].

### Population

The study base comprised people born between 1957 and 1986 (age 29 to 60). The source population was specified in March 2016 using the Regional Primary Care Administrative System and included 200 patients randomly selected from each of the participating GPs’ patient list system irrespective of the postal code of the patient’s residence. The study population was eventually made up of patients who resided in the municipalities of Varde or Haderslev and could be reached by digital mail.

### Recruitment

In April 2016, an initial, digital invitation and informed consent form was sent to the study population. The consent form included participant permission to access specific patient International Classification of Primary Care (ICPC-2) codes and Anatomical Therapeutical Chemical (ATC) codes for information about diagnoses and medical prescriptions, respectively. These records were retrieved from participating GPs’ EPRs. Consenting patients were sent a second digital invitation in September 2016 to fill in a questionnaire on health-risk behaviours, family history of disease, early symptoms of COPD and osteoarthritis. A personal digital health profile was then created for each participant based on the information from their GP’s EPR system and their questionnaire response and accessed on a webpage.

### The personal digital health profile

The personal digital health profile was inspired by research on preventive electronic patient records (EPR) and iteratively co-designed [13–15]. The personal digital health profile provided clear and concise personalised health information, recommendations for further action, including the advice to take up targeted preventive program at the GP or municipality, when relevant, short and concise facts about health-risk behaviour, information about the positive impact of behaviour-change, as well as a personalised list of available and relevant behaviour-change interventions. The aim of the personal digital health profile was fourfold and centered on: 1) motivating and enabling patients who would not otherwise have taken up a targeted intervention as the one offered here, 2) motivating and enabling people with poor self-management skills to take up the targeted intervention, 3) guiding patients with good self-management skills to change their own behaviour, and 4) keeping the healthy, low-risk population from demanding unnecessary health checks from their GP.

### The targeted intervention

Based on EPR and questionnaire information, patients were stratified to one of four groups: Group one consisted of patients who had received treatment for hypertension, hyperlipidaemia, type-2 diabetes mellitus (T2DM), CVD, and/or COPD (as indicated by relevant ICPC-2 and ATC codes) at their GP. These patients were not provided any intervention beyond usual care. Group two included patients who were deemed to benefit from a health check at their GP as determined by three risk algorithms for T2DM, CVD, and COPD [16–18]. As such, these patients were advised to schedule a health check with their GP. The health check comprised a medical examination and a subsequent health counselling session. The health counselling was scheduled as a 30-min session. Group three comprised patients who were not flagged by the risk algorithms, but who had a BMI above 35, and/or reported that they regularly engaged in health-risk behaviour, including daily smoking, high-risk alcohol consumption, unhealthy dietary habits, and sedentary leisure time activities. Patients stratified to group three were advised to schedule a 15-min initial telephone-based counselling session at their municipal health centre (MHC). This could be requested online via their personal digital health profile. If deemed relevant, a one-hour face-to-face behaviour-change counselling session could be scheduled to follow up the initial one. On indication, the patient would be referred to a behaviour-change intervention at the MHC or their GP for medical treatment. Finally, group four consisted of patients with a healthy lifestyle and no need for further intervention.

### Analysis

#### Outcomes

We report on two outcomes. Firstly, we examined attendance or non-attendance at the targeted GP-based health check (Group two). Attendance was defined as having received the medical examination. Secondly, we looked at attendance or non-attendance at the MHC-based counselling session (Group three). Here, attendance was defined as having received the initial telephone-based counselling.

#### Exposures

Exposures included socio-demographic information, mental wellbeing, self-efficacy, health beliefs, health-risk behaviours, BMI, and health-care usage at their GP. Socio-demographics were derived from the national Danish bureau of statistics (Statistics Denmark). Mental well-being, self-efficacy, and health beliefs were derived from a questionnaire presented to all those patients who consented. Data on health-risk behaviours were obtained from the questionnaire which formed the basis for the personal digital health profile. Information on past health checks was retrieved from their GPs’ EPR system. EPR and questionnaire information related purely to consenting patients. All variables were linked by the patients’ unique Danish Personal Identification numbers (CPR) and pseudonymized before being accessible on a secure server at Statistics Denmark.

#### Socio-demographics

Age was categorised into one of two age groups: age 29 to 44 or 45 to 60. Country of origin was determined for the year 2016 and categorised as ‘Danish’ or ‘non-Danish’ origin. Cohabitational status was similarly determined for the year 2016 and categorised as ‘cohabiting’ or ‘single’. Educational attainment for October 2015 was categorised as a binary variable: secondary school and high school, or vocational education and higher education. Employment status for November 2014 was categorised as ‘employed’ or ‘unemployed’. Employed included both employed and self-employed. Unemployed included people on unemployment benefits or who received any form of social welfare or pension. Relative poverty was determined from the households’ annual net income in the years 2013, 2014 and 2015, and defined as below 50% of the median.

#### Health-risk behaviours and biomarkers

Health-risk behaviours and BMI were all dichotomised. Thus, BMI was categorised as either BMI ≥ 30 or BMI < 30. Smoking status was defined as being a current smoker (daily or occasional) or not (stopped or never). As the group of patients with high-risk alcohol consumption comprised few patients, alcohol consumption was split into being above or below low-risk. Low-risk alcohol consumption was set according to Danish guidelines as 7 units/week for women and 14 units/week for men [19]. Physical activity was divided into sedentary or active during leisure time. Active leisure time activity was defined as 4 hours or more of low- to high-intensity leisure time activity (gardening, walking the dog, cycling, etc.) a week. In contrast, sedentary leisure time activity was defined as reading, watching television or other sedentary activities during leisure time. Finally, diet was dichotomised as unhealthy diet or otherwise. Unhealthy diet was defined as a score of four or lower on a 12-point dietary scale [20].

#### Phychological measures

Mental wellbeing was measured using the metrics converted Short Warwick-Edinburgh Mental Wellbeing Scale (SWEMWBS) (range 7 to 35) [21]. Self-efficacy was measured using the generalised self-efficacy scale (range 0 to 40) [22]. Health beliefs were measured on a scale ranging from 4 to 16 and derived from four questions on the perceived importance for health of smoking, diet, alcohol consumption, and physical activity. Each question was phrased as follows” How important for your health do you consider [health-risk behaviour] to be?” with four possible answers (not important, little importance, important, very important) scored from one (not important) to four (very important). The higher the score, the better the mental wellbeing, self-efficacy, and health beliefs.

#### Health-care usage

Previous attendance at the GP was retrieved from their GP’s EPR and defined as having contacted their GP during the two-year period from May 2014 to April 2016 either in-person or by phone. Preventive health checks were defined as having had two or more of the below-mentioned values measured at their GP in the same consultation within a period of 2 years before consenting to the study (May 2014 to April 2016): blood pressure (systolic or diastolic blood pressure), lung function (Forced expiratory volume (FEV1), Forced vital capacity (FVC) or FEV1/FVC), glycated haemoglobin (HbA1c), or lipids (total cholesterol, HDL or LDL).

### Statistical analysis

A one-way ANOVA was used to compare means of numerical exposure variables between the four stratification groups. Chi-square tests were used to compare differences between binary variables. Age, sex, and health-risk behaviours were not compared between the four stratification groups as they were used to determine group belonging. Attendance correlates are presented as crude estimates for all exposure variables (Model 1) as well as estimates adjusted for age and sex (Model 2). We also adjusted exposure variables according to a Direct Acyclic Graph (DAG) causal model of attendance at health checks as conditional interdependencies between exposure variables posed a risk of either residual confounding or collider-stratification bias (Model 3) [23, 24]. We adjusted model 3 in order to avoid collider stratification bias, thus leaving room for possible residual confounding.

The crude estimates were analysed using Chi-square tests and presented as *p*-values. Adjusted estimates were analysed using Poisson regression with robust error variance and presented as incidence rate ratios (IRR), with 95% confidence intervals (CI) of the IRR, and p-values. We chose Poisson regression over logistic regression in order to generate IRR rather than odds ratios (OR) as IRR provides more intuitive estimates in cross-sectional analyses than OR [25]. Moreover, robust error variance was used to reach confidence intervals that are comparable to or superior to those reached from logistic regression [26, 27]. SWEMWBS, self-efficacy, and health beliefs were included in the statistical analyses as continuous variables of the overall score. All other exposure variables were included as categorical variables. Significance level was set at *p* < 0.05.

We used a Chi-square Automatic Interaction Detector (CHAID) analysis to determine the relative strength of the available exposure variables (CHAID command in Stata) [28]. A CHAID analysis essentially uses multiple chi-squared analyses to group categories within each exposure variable into homogenous groups. From the homogenous groups, the CHAID analysis establishes a hierarchy of interactions between exposure variables with the most explanatory exposure variable ranked at the top of the hierarchy. Bonferroni corrections were applied to account for the Type I errors, which may result from multiple uses of chi-square tests. Certain merging, splitting, and stopping rules condition the resulting decision tree. Merging and splitting were given by the selected *p*-value of the Chi-squared analyses and the number of observations in the groups resulting from the Chi-squared analyses. In this study the significance level of merging and splitting (α_merge_, α_split_, and *P*-value) was set at *p* < 0.05. Splitting happened if the number of observations in a node was above 20 (parent node). The CHAID analysis stopped splitting when the number of observations in a sub-category was below a certain threshold. In this study the threshold was set at ten observations (child node). We included two CHAID analyses in this study. One analysis inserted attendance or non-attendance at their GP as the outcome. The other analysis used attendance or non-attendance at the MHC as outcome. All analyses were performed in Stata 14.0.

## Results

**Fig. 1:**
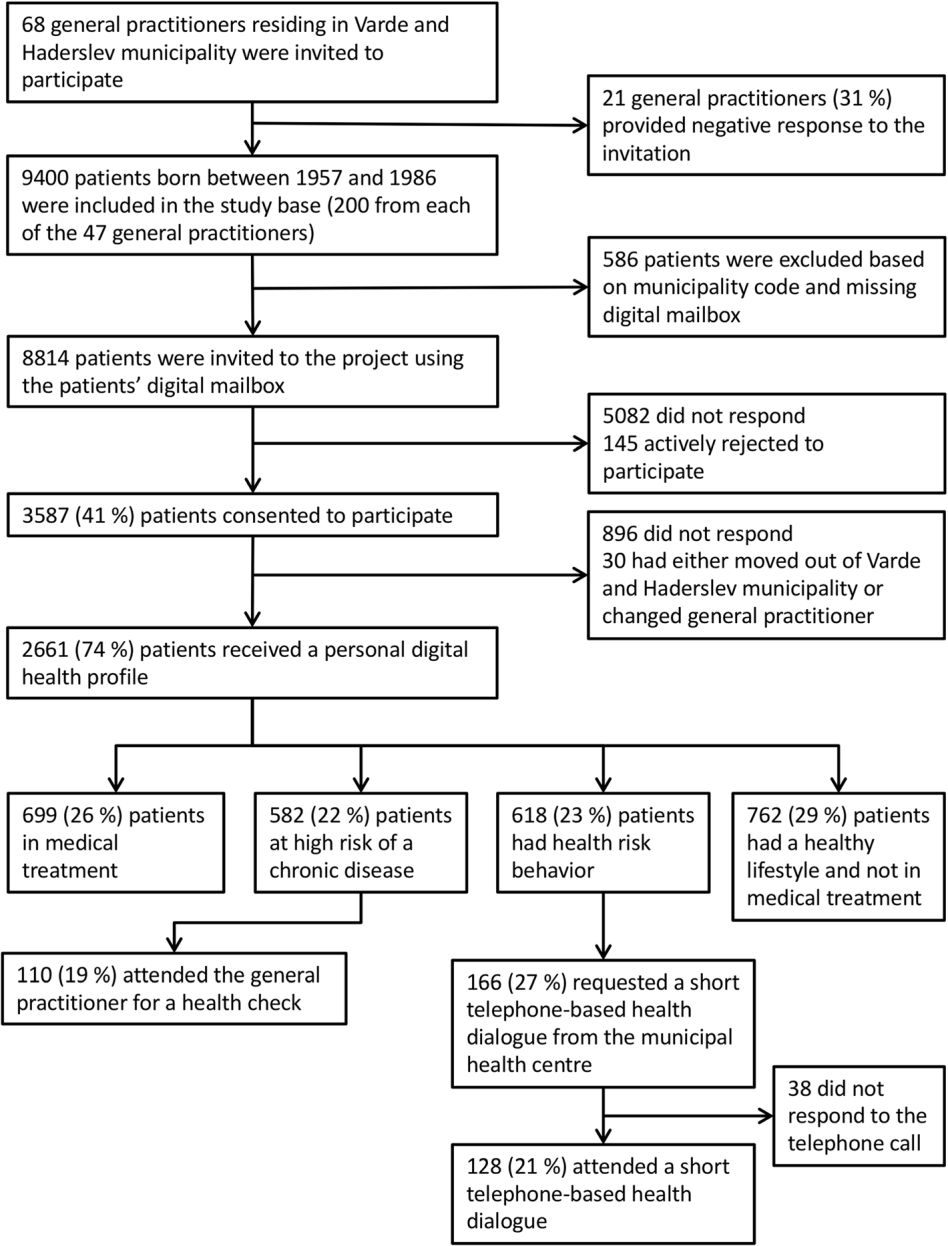
Flow diagram of recruitment, stratification and attendance at the GP-based health check and the initial telephone-based counselling with the MHC

A total population of 9400 patients from 47 GPs (200 from each GP) was randomly selected to form the source population of the TOF pilot study (Fig. 1). Of this population, 586 patients were excluded from the sample as they could not be contacted by digital mail and/or resided outside of the municipalities of Varde or Haderslev. This resulted in a final study population of 8814 patients, of whom 3587 (41%) consented to the study. Of these, 2661 patients (74%) subsequently received a personal digital health profile. Of the 2661 patients, EPR information on 699 patients (26%) indicated that they were diagnosed with, and/or receiving medical treatment for T2DM, CVD or COPD (group one). Based on the questionnaire and EPR information, 582 patients (22%) reached the cut-off of the three risk algorithms and were advised to consult their GP for a health check (group two). Another 618 patients (23%) only exhibited health-risk behaviours and were advised to schedule an initial telephone-based counselling session with their MHC (group three). The rest of the cohort (*n* = 762, 29%) was not at high risk as per the risk algorithms, nor did they engage in health-risk behaviours (group four). Accordingly, they were advised to continue with their healthy lifestyle. Of those patients advised to consult their GP, 110 (19%) did so. Similarly, for patients who exhibited health-risk behaviours, 128 (21%) took the advice to get telephone-based counselling at the MHC.

### Comparisons of the four stratification groups

The mean age of patients who received a personal digital health profile was 45.4 years. Within the group of patients who exhibited health-risk behaviours, the mean age was 42.8 years. Patients who were advised to consult their GP were on average 52.9 years old (Table 1). Overall, 44% of patients who received a personal digital health profile were men. However, men represented only 32% of those patients who did not engage in health-risk behaviour and were not in treatment (group four). Besides, men were overrepresented amongst patients advised to consult their GP (group two), in which 55% were male.

**Table 1:** Descriptive statistics of patient-characteristics and health-care usage in each of the four groups

Patient characteristics	In treatment n (%)	At high risk n (%)	Health-risk behaviour n (%)	No health-risk behaviour and not in treatment n (%)	Total n (%)
	(Group one)	(Group two)	(Group three)	(Group four)	
Total	699 (26)	582 (22)	618 (23)	762 (29)	2661 (100)
Mean age (years)^a^	51.8	52.9	42.8	46.1	45.4
Male^a^	332 (47)	319 (55)	270 (44)	243 (32)	1164 (44)
Danish origin*	673 (96)	567 (97)	596 (96)	731 (96)	2567 (96)
Employed****	575 (82)	521 (90)	538 (87)	710 (93)	2343 (88)
Relative poverty****	99 (14)	61 (10)	120 (19)	71 (9)	351 (13)
Education (Secondary and high school)****	145 (21)	131 (23)	109 (18)	88 (12)	473 (18)
Cohabiting****	545 (78)	458 (79)	479 (78)	659 (86)	2141 (80)
Smokers^a^	140 (20)	83 (14)	237 (38)	0 (0)	460 (17)
Alcohol risk behaviour (above low risk)^a^	92 (13)	72 (12)	73 (12)	57 (7)	294 (11)
Unhealthy diet (dietary score 0–4)^a^	166 (24)	132 (23)	337 (55)	0 (0)	635 (24)
Sedentary behaviour^a^	125 (18)	102 (18)	157 (25)	0 (0)	384 (14)
Body mass index > 30^a^	210 (30)	229 (39)	90 (15)	34 (4)	563 (21)
Self-rated health (Good, very good, excellent)****	555 (79)	506 (87)	536 (87)	728 (96)	2325 (87)
Mean mental well-being (total score)****	23.9	24.4	23.9	25.0	24.2
Mean self-efficacy (total score)****	29.7	30.3	30.1	31.2	30.3
Mean beliefs in a healthy lifestyle (total score)****	13.2	13.1	12.9	14.1	13.3
Health check in past two years^a^	434 (62)	152 (26)	104 (17)	142 (19)	832 (31)

^a^No statistical analysis as the variable was used in the risk stratification

*p > 0.05

**p < 0.05

***p < 0.01

****p < 0.001

Relative poverty was particularly prevalent among patients in the health-risk behaviour group. Furthermore, low educational attainment was particularly prevalent in patients advised to schedule a health check with their GP. Of these, 26% had attended a health check at their GP in the 2 years before receiving their personal digital health profile. Among patients who engaged in health-risk behaviour and who were advised to schedule a behaviour-change counselling session at the MHC, 17% had received a health check during the past 2 years. On the other hand, 19% of patients with a healthy lifestyle and who were not in medical treatment had received a health check during the past 2 years. The prevalence of people of Danish origin did not differ across the four groups.

### Attendance and non-attendance at a GP-based health check (group two)

**Fig. 2:**
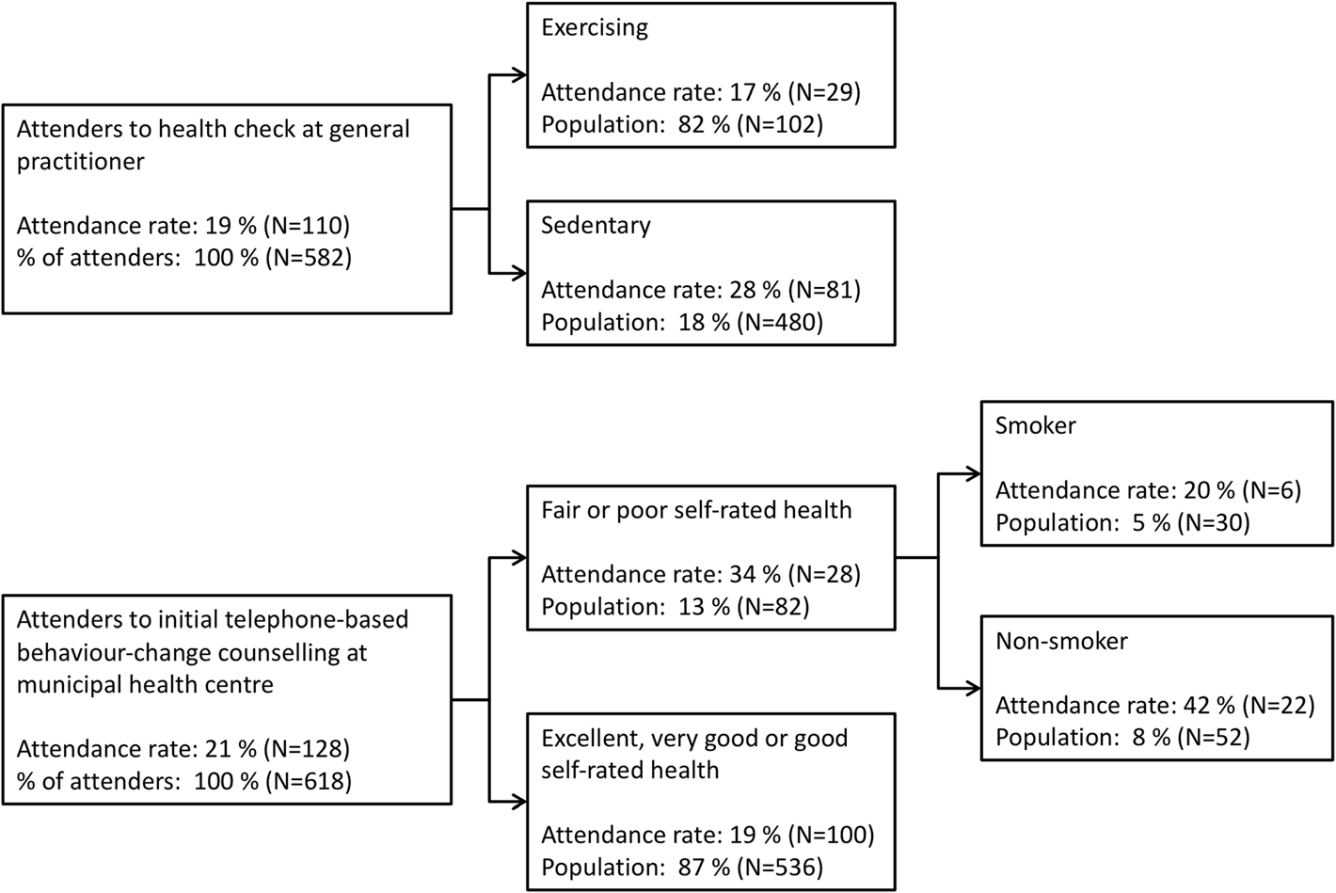
Decision tree analysis of predictors of attendance or non-attendance at the GP-based health check and the initial telephone-based counselling with the MHC

Women and patients with sedentary leisure-time behaviour were more likely to attend a health check at their GP (Table 2). Moreover, lower self-efficacy was associated with a higher likelihood of getting a health check. Age, educational attainment, employment status, relative poverty, smoking status, alcohol consumption, dietary habits, self-rated health, mental wellbeing, beliefs in a healthy lifestyle, and having attended a health check during the past 2 years were not associated with the likelihood of attending a GP health check. The CHAID analysis of GP attendance rates indicated physical activity as the strongest predictor of attendance (Fig. 2). The attendance rate among those with sedentary leisure-time behaviour was 28%, whereas it was 17% among those who engaged in some form of leisure-time exercise.

**Table 2:** Descriptive analysis and three Poisson regression models comparing the incidence rate ratio (IRR) of attendance and non-attendance at a health check at their general practitioner (GP)

Exposures	Patients advised to attend the GP			Model 1 - crude		Model 2 – age and sex adjusted		Model 3 – minimally adjusted	
	Attenders (*N* = 110) n (%)	Non-attenders (*N* = 472) n (%)	Total (*N* = 582) n (%)	IRR	*p*-value	IRR	*p*-value	IRR	*p*-value
Age									
29–44^a^	5 (5)	44 (9)	49 (8)	1 [.;.]		1 [.;.]		1 [.;.]	
45–60	105 (95)	428 (91)	533(92)	2.36 [0.91;6.14]	0.077	2.44 [0.93;6.35]	0.069	2.44 [0.93;6.35]	0.069
Sex									
Male^a^	50 (45)	269 (57)	319 (55)	1 [.;.]		1 [.;.]		1 [.;.]	
Female	60 (55)	203 (43)	263 (45)	1.48 [1.05;2.08]	0.024	1.48 [1.05;2.08]	0.026	1.48 [1.05;2.08]	0.026
Country of origin									
Danish^a^	105 (95)	462 (98)	567 (97)	1 [.;.]		1 [.;.]		1 [.;.]	
Non-Danish	5 (5)	10 (2)	15 (3)	1.81 [0.87;3.79]	0.113	1.83 [0.90;3.74]	0.097	1.86 [0.91;3.80]	0.091
Employment status									
Employed^b^	97 (88)	424 (90)	521 (90)	1 [.;.]		1 [.;.]		1 [.;.]	
Unemployed	13 (12)	48 (10)	61 (10)	1.15 [0.69;1.93]	0.585	1.08 [0.64;1.84]	0.776	1.13 [0.66;1.92]	0.657
Relative poverty^b^	9 (8)	52 (11)	61 (10)	1 [.;.]		1 [.;.]		1 [.;.]	
Other	101 (92)	420 (89)	521 (90)	1.30 [0.69;2.44]	0.409	1.27 [0.68;2.39]	0.455	1.22 [0.66;2.26]	0.524
Educational attainment									
Secondary and high school^b^	22 (20)	109 (23)	131 (23)	1 [.;.]		1 [.;.]		1 [.;.]	
Vocational training and higher education	88 (80)	363 (77)	451 (77)	1.15 [0.75;1.76]	0.517	1.19 [0.78;1.82]	0.428	1.16 [0.76;1.77]	0.499
Cohabitational status									
Cohabiting^b^	86 (78)	372 (79)	458 (79)	1 [.;.]		1 [.;.]		1 [.;.]	
Single	24 (22)	100 (21)	124 (21)	1.04 [0.69;1.56]	0.848	1.06 [0.71;1.59]	0.783	1.06 [0.71;1.59]	0.779
Smoking status									
smoker^b^	15 (14)	68 (14)	83 (14)	1 [.;.]		1 [.;.]		1 [.;.]	
Non-smoker	95 (86)	404 (86)	499 (86)	1.04 [0.64;1.71]	0.863	1.05 [0.64;1.72]	0.841	1.06 [0.65;1.73]	0.819
Alcohol consumption									
above low risk^b^	13 (12)	59 (13)	72 (12)	1 [.;.]		1 [.;.]		1 [.;.]	
Low risk	97 (88)	413 (87)	510 (88)	1.04 [0.62;1.76]	0.871	1.10 [0.65;1.85]	0.727	1.07 [0.63;1.80]	0.803
Diet									
Not unhealthy^b^	29 (26)	103 (22)	132 (23)	1 [.;.]		1 [.;.]		1 [.;.]	
Unhealthy	81 (74)	369 (78)	450 (77)	0.81 [0.56;1.18]	0.278	0.77 [0.48;1.06]	0.093	0.70 [0.47;1.04]	0.075
Physical activity									
Sedentary^b^	29 (26)	73 (15)	102 (18)	1 [.;.]		1 [.;.]		1 [.;.]	
Active	81 (74)	399 (85)	480 (82)	0.61 [0.42;0.88]	0.009	0.61 [0.42;0.88]	0.008	0.62 [0.42;0.90]	0.012
Body mass index									
> 30^c^	63 (57)	290 (61)	353 (61)	1 [.;.]		1 [.;.]		1 [.;.]	
< 30	47 (43)	182 (39)	229 (39)	0.88 [0.63;1.25]	0.482	0.92 [0.64;1.32]	0.632	0.97 [0.67;1.40]	0.860
Health-risk behaviours									
Two or more^b^	12 (11)	32 (7)	44 (8)	1 [.;.]		1 [.;.]		1 [.;.]	
One or none	98 (89)	440 (93)	538 (92)	0.66 [0.40;1.11]	0.117	0.60 [0.35;1.01]	0.054	0.61 [0.36;1.05]	0.073
Self-rated health									
Fair or poor^b^	93 (85)	413 (88)	506 (87)	1 [.;.]		1 [.;.]		1 [.;.]	
Good, very good, excellent	17 (15)	59 (12)	76 (13)	0.86 [0.54;1.38]	0.536	0.89 [0.55;1.42]	0.612	0.88 [0.54;1.45]	0.613
Mental well-being^b^	24.2	24.4	24.4	0.99 [0.94;1.04]	0.573	0.98 [0.94;1.04]	0.552	0.99 [0.94;1.04]	0.575
Self-efficacy^d^	29.4	30.5	30.3	0.96 [0.93;0.99]	0.013	0.97 [0.94;1.00]	0.035	0.96 [0.93;1.00]	0.025
Beliefs in a healthy lifestyle^b^	13.2	13.1	13.1	1.03 [0.94;1.14]	0.503	1.00 [0.91;1.11]	0.942	1.00 [0.91;1.10]	0.997
Appointment at the GP in the past two years									
Yes^b^	97 (88)	389 (82)	486 (84)	1 [.;.]		1 [.;.]		1 [.;.]	
No	13 (12)	83 (18)	96 (16)	0.68 [0.40;1.17]	0.095	0.75 [0.44;1.30]	0.310	0.76 [0.44;1.31]	0.322
Health check in the past two years^a^	79 (72)	351 (74)	430 (74)	1 [.;.]		1 [.;.]		1 [.;.]	
No health check	31 (28)	121 (24)	152 (26)	0.89 [0.61;1.29]	0.546	0.89 [0.62;1.30]	0.557	0.89 [0.62;1.30]	0.557

Model 3 – minimal adjustment

^a^No adjustment

^b^Age, sex, country of origin, education

^c^Age, sex, country of origin, education, occupation, income, cohabitation, smoking, alcohol, diet, exercise

^d^Age, sex, country of origin, education, occupation, income, beliefs in a healthy lifestyle

### Attendance and non-attendance at an initial telephone-based counselling at the MHC (group three)

Non-smokers, patients with BMIs above 30, and patients with poor or fair self-rated health were more likely to attend the initial telephone-based counselling session at their MHC than their smoking, below BMI 30, and good to excellent self-rated health counterparts (Table 3). Similar to attenders at the GP-based health check, lower self-efficacy was associated with a higher likelihood of having an initial telephone-based counselling session. There were no statistically significant associations between age, sex, educational attainment, employment status, relative poverty, alcohol consumption, dietary habits, mental wellbeing, beliefs in a healthy lifestyle, and/or having attended a health check in the past 2 years. The CHAID analysis showed that self-rated health was the strongest overall predictor of attendance, and smoking status was the strongest predictor among patients with fair or poor self-rated health (Fig. 2). The attendance rate was 34% among patients with fair or poor self-rated health, and 19% among those with good to excellent self-rated health. Among patients with fair or poor self-rated health, 20% of smokers attended the initial telephone-based counselling session, whereas 42% of the non-smokers attended.

**Table 3:** Descriptive analysis and three Poisson regression models comparing the incidence rate ratio (IRR) of those patients who took up the initial telephone-based counselling with the Municipal health centre (MHC) and those patients who did not

Exposures	Patients advised to attend MHCs			Model 1 - crude		Model 2 – age and sex adjusted		Model 3 – minimally adjusted	
	Attenders (*N* = 128) n (%)	Non-attenders (*N* = 490) n (%)	Total (*N* = 618) n (%)	IRR	*p*-value	IRR	*p*-value	IRR	*p*-value
Age									
29–44^a^	86 (67)	284 (58)	370 [60)	1 [.;.]		1 [.;.]		1 [.;.]	
45–60	42 (33)	206 (42)	248 (40)	0.73 [0.52;1.01]	0.060	0.73 [0.52;1.02]	0.064	0.73 [0.52;1.02]	0.064
Sex									
Male^a^	59 (46)	211 (43)	270 (44)	1 [.;.]		1 [.;.]		1 [.;.]	
Female	69 (54)	279 (57)	348 (56)	0.91 [0.67;1.24]	0.550	0.93 [0.68;1.27]	0.650	0.93 [0.68;1.27]	0.650
Country of origin									
Danish^a^	121 (95)	475(97)	596 (96)	1 [.;.]		1 [.;.]		1 [.;.]	
Non-Danish	7 (5)	15 (3)	22 (4)	1.56 [0.83;2.95]	0.165	1.62 [0.87;3.01]	0.130	1.62 [0.87;3.01]	0.130
Employment status									
Employed^b^	112 (88)	426 (87)	538 (87)	1 [.;.]		1 [.;.]		1 [.;.]	
Unemployed	16 (12)	64 (13)	80 (13)	0.96 [0.60;1.53]	0.861	0.95 [0.59;1.51]	0.822	0.99 [0.62;1.57]	0.953
Income									
(50% below median income)^b^	22 (17)	98 (20)	120 (19)	1 [.;.]		1 [.;.]		1 [.;.]	
Other	106 (83)	392 (80)	498 (81)	1.16 [0.77;1.76]	0.474	1.20 [0.79;1.83]	0.401	1.21 [0.78;1.82]	0.412
Educational attainment									
Secondary and high school^b^	18 (14)	91(19)	109 (18)	1 [.;.]		1 [.;.]		1 [.;.]	
Vocational training and higher education	110 (86)	399 (81)	509 (82)	1.31 [0.83;2.06]	0.242	1.28 [0.81;2.01]	0.289	1.29 [0.82;2.04]	0.275
Cohabitational status									
Cohabiting^b^	97 (76)	381 (78)	478 (77)	1 [.;.]		1 [.;.]		1 [.;.]	
Single	31 (24)	109 (22)	140 (23)	1.09 [0.76;1.56]	0.641	1.08 [0.75;1.55]	0.675	1.11 [0.77;1.59]	0.579
Smoking status									
Smoker^b^	38 (30)	199 (41)	237 (38)	1 [.;.]		1 [.;.]		1 [.;.]	
Non-smoker	90 (70)	291 (59)	381 (62)	1.47 [1.04;2.07]	0.028	1.44 [1.02;2.02]	0.037	1.45 [1.03;2.04]	0.032
Alcohol consumption									
Above low risk^b^	115 (90)	430 (88)	545 (88)	1 [.;.]		1 [.;.]		1 [.;.]	
Low risk	13 (10)	60 (12)	73 (12)	1.19 [0.71;2.00]	0.517	1.13 [0.67;1.91]	0.640	1.15 [0.68;1.95]	0.613
Diet									
Not unhealthy^b^	78 (61)	259 (53)	337 (55)	1 [.;.]		1 [.;.]		1 [.;.]	
Unhealthy	50 (39)	231 (47)	281 (45)	0.77 [0.56;1.06]	0.110	0.79 [0.57;1.10]	0.165	0.77 [0.56;1.07]	0.116
Physical activity									
Sedentary^b^	34 (27)	123 (25)	157 (32)	1 [.;.]		1 [.;.]		1 [.;.]	
Active	94 (73)	367 (75)	461 (75)	0.94 [0.66;1.32]	0.708	0.96 [0.67;1.36]	0.808	0.99 [0.70;1.41]	0.960
Body Mass Index									
> 30^c^	101 (79)	427 (87)	528 (85)	1 [.;.]		1 [.;.]		1 [.;.]	
< 30	27 (21)	63 (13)	90 (15)	0.63 [0.44;0.90]	0.012	0.65 [0.45;0.95]	0.026	0.62 [0.42;0.91]	0.014
Health-risk behaviours									
Two or more^b^	19 (15)	68 (14)	87 (14)	1 [.;.]		1 [.;.]		1 [.;.]	
One or none	109 (85)	422 (86)	531 (86)	0.93 [0.60;1.43]	0.738	0.96 [0.62;1.48]	0.850	0.95 [0.62;1.47]	0.821
Self-rated health									
Fair or poor^b^	28 (22)	54 (11)	82 (13)	1 [.;.]		1 [.;.]		1 [.;.]	
Good, very good, excellent	100 (78)	436 (89)	536 (87)	0.55 [0.39;0.78]	0.001	0.54 [0.38;0.77]	0.001	0.53 [0.37;0.76]	0.000
Mental well-being^b^	23.4	24.0	23.9	0.96 [0.92;1.01]	0.089	0.97 [0.93;1.01]	0.133	0.97 [0.92;1.01]	0.115
Self-efficacy^d^	29.4	30.2	30.1	0.97 [0.95;1.00]	0.078	0.97 [0.94;1.00]	0.068	0.96 [0.93;0.99]	0.015
Beliefs in a healthy lifestyle^b^	13.1	12.9	12.9	1.05 [0.96;1.13]	0.287	1.05 [0.97;1.14]	0.247	1.05 [0.96;1.14]	0.279
Appointment at the general practitioner in the past two years									
Yes^b^	114 (89)	421 (86)	535 (87)	1 [.;.]		1 [.;.]		1 [.;.]	
No	14 (11)	69 (14)	83 (13)	0.79 [0.48;1.31]	0.361	0.77 [0.46;1.29]	0.327	0.79 [0.47;1.32]	0.364
Health check at the general practitioner in past two years^b^	101 (79)	413 (84)	514 (83)	1 [.;.]		1 [.;.]		1 [.;.]	
No health check	27 (21)	77 (16)	104 (17)	0.76 [0.52;1.10]	0.142	0.73 [0.51;1.06]	0.100	0.73 [0.50;1.05]	0.090

Model 3 – minimal adjustment

^a^No adjustment

^b^Age, sex, country of origin, education

^c^Age, sex, country of origin, education, occupation, income, cohabitation, smoking, alcohol, diet, exercise

^d^Age, sex, country of origin, education, occupation, income, beliefs in a healthy lifestyle

## Discussion

The overall attendance rate was around 20% both for patients who were advised to schedule a health check at their GP (group two) and for patients who were advised to schedule a counselling session at the MHC (group three). Furthermore, our results show that being female, having sedentary behaviour and low self-efficacy were associated to attendance at a health check at their GP (group two). Among patients with health-risk behaviour (group three), non-smokers, patients with fair or poor self-rated health, low self-efficacy, or a BMI above 30 were most likely to attend the initial telephone-based counselling session. We saw no differences in age, educational attainment, employment status, relative poverty, or cohabitational status between attenders and non-attenders at either their GP or MHC. Moreover, as low self-efficacy was associated with higher attendance both at a GP health check and at an initial telephone-based counselling session with an MHC, the personal digital health profile appears to fulfil its aim of motivating and enabling people with low self-management capabilities to attend preventive services. However, it does not meet its other objective of motivating and enabling patients who had not had a health check or visited their GP within the past 2 years. Finally, our results also suggest that health checks provided by the GP as part of their daily clinical practice are currently not only targeted those patients who reached the cut-off of the risk algorithms as one out of five patients with no modifiable health-risk behaviours (group four) had received a health check within the previous 2 years from being invited to the TOF pilot study.

### What is already known

We saw an attendance rate of 20% at both the GP health checks and the initial telephone-based MHC counselling session. The two other known studies that have tested very similar interventions reported an attendance rate to a health check of 36% [8, 11]. However, the attendance rate reported in the study by Van der Meer et al. was unspecific about the online risk assessment and included not only high-risk patients but also patients at low and medium risk [11]. In the study of Van den Brekel-Dijkstra et al., the 36% comprised patients who had completed biometric measures at a research laboratory and not the GP. In the TOF pilot study we did not actively prompt the patients (e.g. with SMS messages or emails) to attend their GP or the MHC during the three-month study period. Prompting has been suggested to increase usage of digital technologies and could possibly have increased the attendance rate [29]. Moreover, pre-booked appointments have previously shown to increase the attendance rate at health checks, but due to technical limitations of the integration of multiple IT-systems, the recommendation to consult their GP or MHC had to be actively heeded by patients (i.e. they had to follow through and book their own appointments) [30]. Additionally, former health-care use has been reported as a reason for non-participation in preventive programs [31]. However, the personal digital health profile was not informed about former health-care usage on who had not had a follow-up on abnormal test results, had not had a health check or had not consulted their GP within the past years.

The study population comprised a selected group of patients who consented to participate in the TOF pilot study and received the personal digital health profile [32]. Our findings on patient characteristics and health-care usage of attenders are, therefore, not directly comparable to the one-step combined risk-assessment and health-check approaches, which are used in many national programmes, including those in Germany, Australia, and South Korea. Nor are our results directly comparable to studies, which make no use of digital feedback on the initial risk assessment such as in the English NHS health check programme [33]. When that said, our results replicate former consistent findings on higher attendance at health checks among non-smokers and women [34–40]. In contrast, the finding that patients with sedentary behaviour are more likely to attend a GP health check differs from earlier studies, which either show no association with physical activity level [11, 34], or higher attendance among physically active patients [41]. Likewise, the finding that patients with a BMI above 30 are more prone to attend a telephone-based counselling session at the MHC, differs from the current evidence that suggests that BMI is not associated with attendance [11, 41], and that the non-obese are more prone to attend [34, 42]. The higher uptake of preventive programs among patients with fair or poor self-rated health differs somewhat from the current sparse and inconsistent evidence. Specifically, our results are at odds with past findings, which indicate that people with higher self-rated health are more likely to take up health checks [41], or that self-rated health is not associated with attendance [34]. Moreover, our findings of no association between attendance and age, and attendance and socio-economic status (SES) such as educational attainment, employment status and relative poverty, differ from previous studies, which show positive correlations between attendance and both age and SES [23, 38, 41, 43].

To our knowledge, self-efficacy has not been examined in relation to attendance at primary preventive programs. However, our results differ from evidence on attendance at rehabilitative services where uptake of cardiac rehabilitation either shows no association or an association with higher self-efficacy [44]. The link between self-efficacy and behaviour (including health behaviour) is represented by the fundamental notion that for an individual to engage in a behaviour, he/she must first be motivated to do so and feel capable of executing the given behaviour [45]. Past research suggests that in a health-behaviour context (including participation in prevention programmes), an individual’s perception of his/her own self-efficacy may be affected by social influences from peers, family, and health professionals, as well as by task-related factors such as planning and goal setting [46, 47]. Thus, we hypothesise two avenues through which the personal digital health profile may have supported the attendance rate among patients with low self-efficacy: 1) By bringing to the fore a variety of health-related behaviour cues. For example, patients may have been motivated to engage in health behaviours (i.e. participation) by their mere interaction with the health professionals who invited them to participate in the study, generated their personal digital health profile, and were readily available for further support. 2) In a more direct manner, the concrete advice and guidance that patients were given in their personal digital health profile about why, how, and where to get a health check or behaviour-change counselling may also have endowed them with the knowledge to complete these tasks. That is, continuously showing participants the way to take preventive action step by step, may have framed the task of getting a health check as surmountable and manageable. By contrast, without all this information and support from the personal digital health profile, individuals with perceived low self-efficacy may have viewed the same task as too difficult to complete. As such, the interaction with health professionals combined with the information about one’s own health (risk assessment) may have motivated people to do something, whereas the guidance, which the personal digital health profile offers, may have facilitated the individual’s translation of that motivation into action (enabled them).

Further research is warranted to test this hypothesis, as well as research on the association of personal digital health profiles with motivation and enablement among those with e.g. low self-efficacy.

### What this study adds

In a related paper we showed that the response to the first digital invitation and subsequent consent to participate in the TOF pilot-study was skewed towards higher uptake among women, the older people, and patients with higher SES [32]. However, this study suggests that a personal digital health profile may help foster a more equitable uptake of preventive programs in the primary care sector – especially among patients with lower self-efficacy and fair to poor self-rated health.

Previous studies on prevention in the primary care sector have mostly focused on either health checks or behaviour-change counselling [48–50]. However, recent studies report on interesting, though inconclusive, results on linking primary care and community health [51, 52]. Our findings suggest that personal digital health profiles may be a valuable asset in the recruitment of patients to preventive programs across primary care providers.

However, the evidence is sparse on the reach and effects of personal digital health profiles used for preventive programs in the primary care sector, indicating that further research on personal digital health profiles, as part of a comprehensive approach to preventive programs in the primary care sector, is needed.

### Strengths and limitations

A major strength is the use of high quality national health and social registries with very few missing observations combined with complete and comprehensive self-reported patient information and EPR information from their GP [53, 54]. Another strength is the randomized source population. However, low and skewed initial participation led to a study population with more women, and higher average SES and age than in the randomized source population [32].

As much as a digital approach to the patient may make use of behaviour-change techniques to increase usage, it may also be a disadvantage: the health profile is only available on the web and for people with the required access, devices, skills and literacy. A purely digital approach may be beneficial for some groups of patients, but not for others, and should be supplemented by other options, such as waiting-room administered systems and outreach [55].

## Conclusions

A personal digital health profile shows some promise as a component in a step-wise approach to preventive programs in primary care. Specifically, personal digital health profiles fulfil the aim of motivating people with low self-efficacy to attend, however not of motivating patients who had not had a health check or visited their GP during the past years. Further, women and patients with fair or poor self-rated health, a BMI above 30, low self-efficacy, sedentary behaviour and non-smokers were more likely to take up preventive programs in the primary care sector following a personal digital health profile. The uptake was similar across age, SES and health-care usage strata.

## Data Availability

The data that support the findings of this study will be available from Statistics Denmark but restrictions apply to the availability of these data, which will be used under license for the current study, and so are not publicly available. Data will however be available from the authors upon reasonable request and with permission of Statistics Denmark.

## Abbreviations

ATC: Anatomical Therapeutic Chemical Classification System
BMI: Body mass index
CHAID: Chi-squared Automatic Interaction Detection
COPD: Chronic Obstructive Pulmonary Disease
CPR: Danish Personal Identification number
CVD: Cardiovascular Disease
DAG: Direct Acyclic Graph
EPR: Electronic Patient Record
FEV1: Forced expiratory volume
FVC: Forced vital capacity
GP: General Practitioner
HbA1c: Glycated haemoglobin
HDL: High-density lipoprotein
ICD-10: International Classification of Diseases 10th Edition
ICPC-2: International Classification of Primary Care 2nd Edition
IRR: Incidence Rate Ratio
KEU: The joint Quality and educational committee of the Region of Southern Denmark
LDL: Low-density lipoprotein
MHC: Municipal Health Centre
MRC: Medical Research Council
NHS: National Health Services
OECD: Organisation for Economic Co-operation
PLO: Danish General Practitioners Union
SES: Socio-Economic Status
SWEMWBS: Short Warwick-Edinburgh Mental Wellbeing Scale
T2DM: Type-2 Diabetes Mellitus
TOF: Project Early Detection and Prevention
